# Association between Diet Quality and Sarcopenia in Older Adults: Systematic Review of Prospective Cohort Studies

**DOI:** 10.3390/life11080811

**Published:** 2021-08-10

**Authors:** Eun-Hee Jang, Ye-Ji Han, Seong-Eun Jang, Seungmin Lee

**Affiliations:** Department of Food and Nutrition, Sungshin Women’s University, Seoul 01133, Korea; 2201957752@sungshin.ac.kr (E.-H.J.); 220216020@sungshin.ac.kr (Y.-J.H.); 220206127@sungshin.ac.kr (S.-E.J.)

**Keywords:** sarcopenia, muscle mass, muscle strength, diet, diet quality, systematic review, epidemiology

## Abstract

(1) Background: Nutrition is a key determinant of sarcopenia in later life. (2) Methods: A systematic review of prospective cohort studies examining association of diet quality with muscle mass (MM), muscle strength (MS) or physical performance (PP) among older adults was conducted. A total of 22,885 results were obtained from a literature search in MEDLINE via PubMed and EMBASE up to November 2020. Inclusion criteria included diet quality assessment via dietary indices or statistical approaches, a sample of adults aged 45 years and over at baseline in a longitudinal study design. (3) Results: Of the 22,885 cohort studies, 14 studies were eligible. Meaningful results were obtained for the Mediterranean diet and Nordic diet regarding the decrease of sarcopenia risk, however results from non-European countries were inconsistent. In addition, due to the insufficient number of studies on Japanese Food Guide Spinning Top (JFG-ST), dietary variety score (DVS), and dietary quality index-international (DQI-I), effectiveness was difficult to prove. Studies using factor analysis to examine dietary patterns suggested that the risk of sarcopenia is increased with a high in saturated fat diet such as westernized pattern etc. (4) Conclusion: In this systematic review it was found that various diet qualities are meaningful to a decreased risk of sarcopenia.

## 1. Introduction

Sarcopenia, a common and major problem in older adults, is associated with a decrease in muscle mass, muscle strength, and physical performance. The European Working Group on Sarcopenia in Older People (EWGSOP) published a globally accepted definition of sarcopenia in 2010 [1], and an ICD-10-CM code for sarcopenia was established in 2016 [2]. The Working Group met again (EWGSOP2) in 2018 to review the recently accumulated scientific evidence and the definition of sarcopenia was updated [3].

Previous studies have reported varying prevalence of sarcopenia from 9.9 to 40.4%, depending on the diagnostic criteria [4]. Sarcopenia affects the status of health, leading to heavy personal and social economic burdens if not treated [5,6], therefore optimal care for sarcopenia is essential. However, treatment of sarcopenia is difficult due to the complex pathophysiology involving age-related changes such as neurodegeneration, reduction in anabolic hormone, dysregulation of cytokines, and modification of the inflammatory state [1,7].

Several cohort studies have reported that nutritional intervention can improve muscle mass, muscle strength, and physical performance. Large epidemiologic cohort studies have reported an association of the risk of sarcopenia and the intake of protein [8,9], meat [10], dairy products [11], and fruits and vegetables [12,13]. Most previous studies focused on single nutrients or foods, thus identification of complex interactions and synergistic effects is difficult. Study of the overall diet is important in complementing the limitations of previous studies.

There are three major methods for study of the overall diet: score or diet quality indicators, principal components or cluster analysis, a combination of biological pathways, and underlying dietary data [14]. These different approaches are complementary, and determining the best one is difficult. Therefore, this review included all studies without restriction of methodologies for assessment of the overall diet.

Reduced dietary intake due to an impaired peripheral satiety system, decreased taste or smell functions, and increased cytokine production [15] cause difficulty in meeting dietary recommendations for older adults. In addition, adherence to a healthy diet in elderly persons may vary individually according to underlying social and psychological factors throughout life [16]. Long-term poor diet quality based on these factors could have a serious effect on health status.

However, little is known about the effects of overall diet quality on sarcopenia. A systematic review including 12 prospective cohort studies was recently conducted to investigate the effect of overall dietary intervention to improve muscle mass, muscle strength, or physical performance in older people [17]. The study concluded that there is a lack of longitudinal evidence for the relationship between diet quality and sarcopenia and that further studies are needed. Therefore, the aim of this systematic review is to update the longitudinal evidence, including studies published up to November 2020.

## 2. Materials and Methods

The systematic review was conducted based on a protocol generated in accordance with the Preferred Reporting Items for Systematic Reviews and Meta-Analyses (PRISMA) statement [18].

### 2.1. Data Sources and Search Strategy

The literature searches were performed for articles published from database inception to the current data on 30 November 2020. MEDLINE via PubMed and EMBASE was searched using a combination of search terms related to sarcopenia, older adults, and overall diet. The search was limited to publications in English and studies conducted in humans. The search strategy is shown in Supplementary Table S1. References of included studies and relevant review papers were reviewed for additional citations.

### 2.2. Study Selection

**Figure 1:**
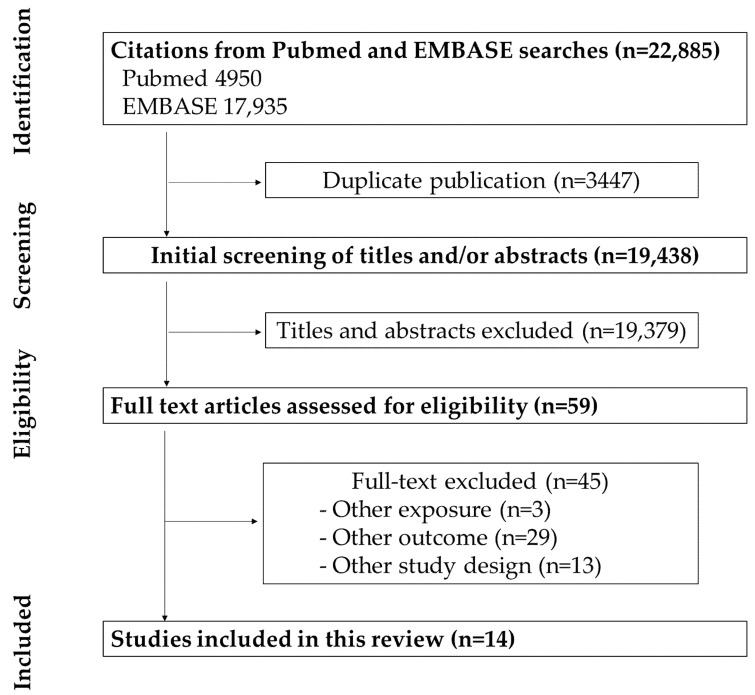
PRISMA flow diagram.

The process of identifying included studies is shown in Figure 1. A total of 22,885 studies were initially searched using the Medline and Embase databases; 3447 were duplicate articles. The remaining 19,438 papers were screened for title and/or abstract; 19,337 were excluded. An additional 59 papers were excluded based on assessing the eligibility of the full articles. Finally, 13 studies were included in the review. All study selection procedures were performed by three authors (E.-H.J., Y.-J.H., and S.-E.J.).

Prospective cohort studies reporting on a relationship between diet quality and sarcopenia in older adults that met the criteria below were included. The inclusion criteria were as follows: (1) prospective cohort studies with full-text availability in English; (2) mean age of study participants ≥40 years at baseline; (3) including healthy adults without diseases other than overweight or type 2 diabetes; (4) overall diet quality as an exposure; (5) at least one outcome repeated measure of sarcopenia (muscle mass, muscle strength, physical function). The exclusion criteria were as follows: (1) the study only reported “health score” combining diet quality with other lifestyle/behavior; (2) the study evaluated single foods or nutrients only; (3) the study included medical records or a self-reported diagnosis for a clinical condition and did not assess current health using an assessment tool or questionnaire.

### 2.3. Data Extraction and Quality Assessment

Information on the number and age of participants, details of interventions, outcome measures, and adverse events were extracted from each study. A quality assessment was evaluated using the Risk of Bias for Nonrandomized Studies (RoBANS) tool for cohort studies as “low”, “high”, and “unclear” risk of bias [19]. RoBANS, which was developed in Korean for evaluation of non-randomized interventional research, is shown in Table 1.

## 3. Results

### 3.1. Description of the Studies

In this study, 14 studies on the association between overall diet and sarcopenia were reviewed. Included studies were conducted in Italy [20,22], the United States of America (USA) [21], Spain [23,25], China [28], Finland [27,29,31], Japan [24,30,33], and the United Kingdom (UK) [26,32]. Studies were published since 2011; the exact publication years were as follows: 2011 [20]; 2012 [21,22]; 2014 [23]; 2015 [24,25]; 2016 [26,27,28]; 2017 [29,30]; 2018 [31]; 2020 [32,33]. Studies were grouped according to the tools used for assessment of diet quality. Studies evaluating adherence to diets known to be healthy were assessed on the Mediterranean diet score (MDS) [20,21,22,23,28,31,33], the Nordic diet score (NDS), also known as the Baltic sea diet (BSD) score [27,29,31], the Japanese diet, and the dietary approaches to stop hypertension (DASH) [33]. Other studies used the following diet quality indicators as assessment tools: the dietary quality index-international (DQI-I) [28] and the dietary variety score (DVS) [24,30]. Regarding the last three studies of factor analysis, one compared a westernized pattern with low diet quality including foods with high fat content, with a prudent pattern with high diet quality including foods with olive oil, vegetables, and fish [25]. Another study divided dietary patterns such as ‘high red meat’, ‘low meat’, and ‘high butter’ [26], while another study classified diet as ‘low red meat’, ‘traditional British’, and ‘low butter’ [32].

Dietary intake for evaluation of adherence to a particular diet was examined using various tools. These included the following: (1) a validated food frequency questionnaire (FFQ); (2) a modified version of the validated FFQ, (3) a validated brief dietary history questionnaire; (4) the dietary recall method; (5) a validated computerized diet history; and (6) an interview. Similarly, muscle mass (MM), muscle strength (MS), and physical performance (PP), which are components of the sarcopenia definition, were measured using various methods. (1) MM was evaluated by whole body dual-energy X-ray absorptiometry (DXA) scans or whole-body bioelectrical impedance analysis (BIA), and in some studies skeletal muscle index (SMI) was calculated by dividing the muscle mass by squared height; (2) MS was evaluated using hand grip strength or knee extension strength using a dynamometer; (3) PP was evaluated using walking speed or objective assessment tools such as a Timed Up-and Go (TUG) test, a short physical performance battery (SPPB), and Senior Fitness Test (SFT). SPPB includes walking speed 10 m, chair rises in 30 s and one leg stance performance. The SFT battery consists of five measurements of physical fitness: the number of chair stands during 30 s; the distance walked in 6 min; the number of arm curls during 30 s; chair sit and distance between fingers and toe; and back scratch (distance between extended middle fingers). Finally, some studies (4) evaluated sarcopenia according to the EWGSOP/AWGS algorithm (Table 2), after measurement of MM, MS, and PP using the various methods presented above.

Study results are presented in the odds ratio (OR) and hazard ratio (HR) with 95% confidence intervals (95% CI), or mean difference, GEE model estimates, beta coefficients, and standard error (SE). *P* values are provided when available.

The risk of sarcopenia according to each diet quality was reviewed. When two or more diet qualities were evaluated, the study was described repeatedly in each category. When both cross-sectional and prospective data were analyzed, only the results using the prospective data were described.

#### 3.1.1. Mediterranean Diet

Seven studies on the association between the Mediterranean diet and sarcopenia are shown in Table 3. Sample sizes ranged from 253 [31] to 2948 [28] and the mean age of the study participants was 67.4 [31] to 74.6 years [21]. One study included only women [31], one study included only men, and five studies investigated both women and men [20,21,22,23,28,33].

In prospective studies of Mediterranean diet adherence and MM, one study reported no association with SMI measured using BIA [33], whereas another study including only women found a significant association with the total body lean mass (LM) measured by whole body DXA and the relative skeletal muscle index (RSMI) [31]. Adherence to a Mediterranean diet and MS showed a significant association with the weakness as the lowest quintile of grip strength in only one [23] of four cohort studies [21,26,29]. In the case of PP, in a 9-year follow-up study, high adherence to the Mediterranean diet slowed the decrease in walking speed, and the risk of developing new mobility disability [20]. Similarly, various studies reported an association with adherence to a Mediterranean diet and low walking speed, usual 20-m walking speed, and rapid 20-m walking speed [21,22,23], whereas three studies including a prospective study of only women found no association [23,26,29]. Finally, the two studies reported no relationship between the Mediterranean diet score and sarcopenia [28,31]. Given the different results of the association of the Mediterranean diet with sarcopenia and its indices in the above various cohort studies, we report that there is a conflicting level of evidence.

#### 3.1.2. Diet Quality Other Than the Mediterranean Diet

The association between Sarcopenia and various diet qualities, except the Mediterranean diet, is shown in Table 4. The NDS was reviewed in two studies analyzing data from the same cohort study and found an association between higher NDS and higher PP and MS in women only. For MM, no association was observed in both men and women [27,29]. Another study analyzing 259 women found that a higher NDS resulted in less MM and PP loss over time but was not associated with MS [31]. In a longitudinal study of Japanese Food Guide Spinning Top (JFG-ST), modified JFG-ST (mJFG-ST), and DASH, only men with higher JFG-ST adherence other than mJFG-ST and DASH were more likely to have higher SMI [33].

A prospective study [30] examining the relationship between DVS and sarcopenia provided strong support that a higher DVS in the elderly leads to an increase in handgrip strength and general gait speed. In contrast, a cohort study of 575 women reported no relationship between DVS and knee extension strength [24]. A study examining the association of DQI-I score and three dietary patterns using factor analysis (‘vegetables-fruits’ diet, ‘snacks-drinks-milk products’ diet, and ‘meat-fish’ diet) with sarcopenia in Chinese community-dwelling older people found no association between dietary patterns and sarcopenia [28].

In the Spanish cohort study, ‘westernized pattern’ was defined as the high consumption of refined bread, whole dairy products, and red and processed meat, as well as the low intake of whole grains, fruit, low-fat dairy, and vegetables by factor analysis. One study reported that greater adherence to a westernized pattern resulted in slower walking speed [25]. A factor analysis study conducted in the UK found an association between dietary patterns ‘high red meat’ and ‘high butter’ with decreased muscle strength and physical performance [26]. Another study of dietary patterns using the same cohort data reported an increased risk of sarcopenia regardless of protein status in the ‘traditional British’ diet (in high butter, red meats/meat dishes, gravy, potatoes, vegetables, sweets/desserts, and the highest intake of fat and total energy) compared with the ‘low butter’ diet (in high unsaturated fat spreads/oils, fiber, and the highest % energy from protein and starch) [32].

## 4. Discussion

In this study a systematic review of cohort studies on the relationship between diet quality and risk of sarcopenia in older adults was conducted. The various methods used to examine diet quality include score or diet quality indicators, principal components, and cluster analysis. The score or diet quality indicators covered in this review include the Mediterranean diet, NDS, DASH, JFG-ST, mJFG-ST, DVS, and DQI-I. Articles on various dietary patterns extracted by factor analysis, such as ‘prudent’, ‘westernized’, ‘vegetables-fruits’, ‘snacks-drinks-milk products’, ‘meat-fish’, ‘high red meat’, ‘low meat’, ‘high butter’, ‘low red meat’, ‘traditional British’, and ‘low butter’ are also included. As discussed above, the articles defining a ‘healthy diet’ included various methods of measuring dietary patterns. Therefore, in reporting the results, this research grouped the assessment methods of diet quality and suggested association with sarcopenia for each group.

A lower rate of protein synthesis and less protein intake is observed in older adults compared with younger adults. Additionally, the pathogenesis of sarcopenia may be influenced by oxidative stress and inflammation. Previous studies have demonstrated that an adequate amount of protein and certain nutrients with antioxidant and anti-inflammatory functions including vitamin D, selenium, magnesium, and omega-3 fatty acids could have a protective effect against decline in muscle mass, strength, and function associated with aging [35].

The Mediterranean diet is high in vegetables, legumes, fruits, nuts, grains, and fish, particularly olive oil, but low in red meat and poultry, and appropriate in dairy product and alcohol in the form of wine. Higher adherence to the traditional Mediterranean diet significantly reduces the risk of chronic diseases such as cardiovascular disease, cancer, and diabetes, total mortality, as well as low cognitive function in healthy older adults [36,37,38,39,40]. Some nutrients (vitamins, minerals, and omega-3 fatty acids, etc.) with antioxidant and anti-inflammatory functions derived from food sources such as fish, vegetables, and fruits may be related to the protective effect against sarcopenia. However, because the Mediterranean diet is a regional diet, the beneficial effects of adherence to the Mediterranean diet are controversial in non-Mediterranean regions due to differences in food availability, eating and lifestyle habits [41]. Studies conducted in European countries (two in Italy, one in Spain, and one in Finland) reported a significant association between the Mediterranean diet and sarcopenia. Among these studies, one study conducted in Finland suggested the risk of sarcopenia as an outcome, and the others reported MM, MS, or PP which are the components of the sarcopenia definition [20,21,23,26]. In other non-European countries, associations were reported in a study conducted in the United States [21], but no association was reported in studies conducted in China and Japan [28,33].

The Nordic diet was developed based on the opinions of experts in the fields of human nutrition, gastronomy, environmental issues, food culture and history, sensory science in 2010 based on the Danish food culture [42]. This diet is similar to the Mediterranean diet, however it differs in the use of canola oil high in omega-3 fatty acid instead of olive oil. Therefore, as with the Mediterranean diet, nutritional components derived from foods such as vegetables and fruits, fish, and canola oil are expected to be beneficial in sarcopenia. Also, the same effect of the Nordic diet cannot be proven in non-Nordic regions. Studies on NDS have only been reported since 2016, and only three studies have been reported in Finland [27,29,31]. Two of these studies are based on the same cohort data, thus providing sufficient evidence is difficult. The results of the study were significant in PP, MM, and MS, however, most of the results were limited to women, which seems to suggest potential sexual dimorphism between males and females, and further study is needed.

JFG-ST was based on six food groups (i.e., grain dishes, vegetable dishes, fish and meat dishes, milk, fruit, snacks, confection, and beverages) [43]. Greater adherence to JFG-ST could improve muscle mass in the elderly, but MD, DASH, and mJFG-ST showed no association with MM, MS and PP [33]. In response, the researchers in this study reported that there may be inconsistencies in the results due to various eating habits according to region.

In addition, three studies were conducted on the dietary index score and sarcopenia in Asia, two of DVS and one of DQI-I. Two studies on DVS were conducted in Japan. An earlier study conducted in 2015 reported no association with sarcopenia [24], but the prevention of MS and PP was reported in the high DVS group in a study conducted in 2018 [30]. DVS is a score for a variety of food composition, therefore confirming the effect of specific foods or nutrients on sarcopenia is difficult. However, in this study, high DVS resulted in higher consumption of healthy foods such as fruits, vegetables, and fish. They also noted that it would be worthwhile to conduct a study on sarcopenia and DVS, as a previous cross-sectional study [44] confirmed the prevention effect of the decline of MS and PP on high DVS. Only one Chinese study on DQI-I was reported in 2016, which reported no significant effect [28].

Finally, in studies on dietary pattern using factor analysis, high adherence to a westernized pattern characterized by high intake of refined cereals, whole dairy, and red and processed meat resulted in a significant decrease of PP in a Spanish study [25]. In two studies by the same researcher conducted in the UK, a meal composition with a high intake of red meat and butter reduced MS and PP and increased the prevalence of sarcopenia compared to a relatively healthy diet consuming unsaturated fat spreads, olive oil, and other plant-based fats [26,32]. According to the type and quality of dietary fat, diets that are especially high in saturated fat may affect sarcopenia due to increased catabolism, such as inflammation and oxidative stress, and fat accumulation in aged muscle [45]. On the other hand, a cohort study conducted in China, which classified a diet into three types of high intake of vegetables-fruits, snacks-drinks-milk products, and meat-fish, reported no association with sarcopenia [28].

Our study has several limitations that should be considered. Although validated tools were used for most diet assessment methods, measurement errors cannot be excluded. The method used to evaluate the same diet quality differs between studies and should be considered when making comparisons. Although our study attempted to report the relationship between diet quality and risk of sarcopenia, it had not been a long time since sarcopenia was defined by a global consensus; a single evaluation of MM, MS, and PP was performed respectively instead of the risk of sarcopenia in most of the articles. In addition, because tools for measurement of PP include walking speed, TUG, SPPB, etc., making an objective comparison was difficult because the tools for measuring outcomes differed for each study. For the same reason, there was a greater insufficiency of longitudinal data to evaluate the effectiveness. Nevertheless, in our study only the results of longitudinal data analysis were included to report causality. If the association was observed only in cross-sectional data obtained in the same study, it was reported as no association at all in this review. It should be considered that the inclusion of these possibly relevant studies could have led to potentially different conclusions. In the case of sarcopenia in the elderly, the relationship with diet quality may weaken over time, therefore caution is required in interpretation. Finally, there may be gray literature and papers on these associations. Despite these limitations, this review has several strengths. To the best of our knowledge, this is the first systematic review of only cohort studies of the association between sarcopenia and overall diet quality. There is no concern about reverse association by reviewing the results of only longitudinal studies between overall diet quality and risk of sarcopenia. Our findings provide the basis for further investigations to determine whether there is a causal relationship between diet quality and sarcopenia through systematic review including factor analysis as well as region-based diet quality and diet quality index. This research area is relatively new, therefore, establishment of a standard definition for diet quality focused on sarcopenia and high-quality cohort studies using consistent diet quality measurements is needed. In addition to high-quality longitudinal studies, randomized controlled trials designed to evaluate dietary interventions as a strategy for prevention and/or management of sarcopenia should be conducted, as diets may vary over time due to social and cultural factors. After conduct of a sufficient number of studies, a meta-analysis should be conducted for a more thorough evaluation of the level of influence of diet quality.

## 5. Conclusions

In conclusion, the only diet quality significantly correlated with sarcopenia is the Mediterranean diet, the most studied until recently. However, significant results were obtained mostly in Mediterranean countries, the number of studies was insufficient, and the results were inconsistent in non-Mediterranean countries. The Nordic diet is similar to the Mediterranean diet, therefore a beneficial effect is expected; further studies are needed due to limited NDS research results regarding women in Finland. In addition, in studies on JFG-ST, DVS, and DQI-I, effectiveness was difficult to prove due to an insufficient number of studies. In addition, results of factor analysis of various dietary patterns suggested that saturated fat increases the risk of sarcopenia depending on the composition and amount of fat. Results of longitudinal analysis from different countries were not always similar due to differences in food availability and methods of food preparation from region to region. Conduct of high-quality cohort studies and randomized controlled trials for evaluation of diet quality as a strategy for prevention and/or management of sarcopenia is needed. A meta-analysis could be conducted after conduct of a sufficient number of studies.

## Figures and Tables

**Table 1:** Risk of bias of included cohort studies on the impact of dietary patterns on sarcopenia risk.

Study ID	Selection of Participants (Selection Bias)	Confounding Variables (Confounding Bias)	Measurement of Exposure (Performance Bias)	Blinding of Outcomes Assessment (Detection Bias)	Incomplete Outcome Data (Attrition Bias)	Selective Outcome Reporting (Reporting Bias)
Y. Milaneschi et al., 2011 [20]	Low	Low	Low	Low	High	Low
D.R. Shahar et al., 2012 [21]	Low	Low	Low	Low	Low	Low
S.A. Talegawkar et al., 2012 [22]	Low	High	Low	Low	High	Low
L.M. León-Muñoz et al., 2014 [23]	Low	High	Low	Low.	Low	Low
N. Kojima et al., 2015 [24]	Low	Low	Unclear	Low	High	Low
L.M. León-Muñoz et al., 2015 [25]	Low	Low	Low	Low	High	Low
A. Granic et al., 2016 [26]	Low	Low	Low	Low	High	Low
M.M. Perälä et al., 2016 [27]	Low	High	Low	Low	High	Low
R. Chan et al., 2016 [28]	Low	High	Low	Low	High	Low
M.M. Perälä et al., 2017 [29]	Low	Low	Low	Low	High	Low
Y. Yokoyama et al., 2017 [30]	Low	Low	Low	Low	High	Low
M. Isanejad et al., 2018 [31]	Low	Low	Low	Low	Low	Low
A. Granic et al., 2020 [32]	Unclear	Low	Low	Low	High	Low
C.H. Huang et al., 2020 [33]	Low	Low	Low	Low	High	Low

**Table 2:** Definition and cut-off points of sarcopenia according to the EWGSOP1/EWGSOP2/AWGS algorithm.

	EWGSOP1 [1]	EWGSOP2 [3]	AWGS [34]
Definition	Diagnosis is low MM plus low MS or low PP	Possible sarcopenia is identified by low muscle strength. The diagnosis is confirmed by low muscle mass or quality. Sarcopenia is considered severe in the presence of low muscle mass, low muscle strength, and low physical performance.	Diagnosis is low skeletal muscle mass plus low muscle strength and/or physical performance
Low MM	SMI by DXA, men: <7.26 kg/m^2^; women:<5.5 kg/m^2^. SMI by BIA, men: 8.87 kg/m^2^; women: 6.42 kg/m^2^	ASM, men: <20.0 kg; women: <15.0 kg. ASM/height^2^, men: <7.0 kg/m^2^; women: <5.5 kg/m^2^	ASM by DXA, men: <7.0 kg/m^2^; women: <5.4 kg/m^2^. ASM by BIA, men: <7.0 kg/m^2^; women: <5.7 kg/m^2^
Low MS	Handgrip strength, men: <30 kg; women: <20 kg	Handgrip strength, men: <27 kg; women: 16 kg. Chair stand, >15 s for five rises.	Handgrip strength, men: <28 kg, women: <18 kg
Low PP	Gait speed: >0.8 m/s	Gait speed: >0.8 m/s; SPPB: ≤8; TUG: ≥20 s; 400 m walk test: Non-completion or ≥6 min for completion	6-meter walk: <1.0 m/s; 5-time chair stand test: ≥12 s; SPPB: ≤9

MM: muscle mass; MS: muscle strength; PP: physical performance; SPPB: Short Physical Performance Battery; TUG: Timed Up-and Go; ASM: Appendicular skeletal muscle mass.

**Table 3:** Summary of associations between sarcopenia and Mediterranean diet.

Author (Year), country	Cohort, Period	Study Participants	Dietary Assessment	Sarcopenia Assessment	Covariates	Findings
Y. Milaneschi et al. (2011), Italy	InCHIANTI Study Follow-up period: nine years (1998–1999 to 2007–2009)	Community-living persons (≥65 years) randomly selected from a population registry in Tuscany, Italy Sample size: 935 (415 men, 520 women) Age, Mean (±SD): 74.1 (6.8)	**Tool:** FFQ **DQ:** MDS, ranged from 0 to 9, categorized into three groups: low adherence (MDS ≤ 3); medium adherence (MDS 4–5); high adherence (MDS ≥ 6)	**PP:** SPPB	Age, sex, energy intake, BMI, MMSE score, physical activity, ADL and IADL disabilities, depressed mood, number of chronic diseases and medications	**PP:** SPPB scores according to the MDS groups (the high as compared with the low), GEE model estimates 0.86 (SE = 0.21, *p* < 0.001) at 3-year follow up; 1.09 (SE = 0.29, *p* = 0.0002) at 6-year follow up; GEE model estimates 0.90 (SE = 0.41, *p* = 0.03) at 9 year follow up
						In participants without ADL disabilities and SPPB ≥ 10, **PP:** Mobility disability (SPPB ≤ 9) according to the MDS groups, low: referent, high: HR 0.71 (95% CI = 0.51–0.98); per ↑ 2 point MDS: HR 0.86 (95% CI = 0.74–0.99)
D.R. Shahar et al. (2012), USA	Health ABC Study Follow-up period: eight years (recruited in 1997–1998, started at year 2)	Well-functioning individuals aged 70 and older Sample size: 2225 (1114 men, 1111 women) Age, Mean (±SD): by MedDiet score group, Low 74.5 (2.8); Middle 74.6 (2.9); High 74.6 (2.7)	**Tool:** Modified FFQ, 108 items **DQ:** MedDiet score, divided into three levels: low adherence (0–2); medium adherence (3–5); high adherence (6–9)	**PP:** Walking speed, usual and rapid assessed over a 20-m course	Age, race, sex, education, site, smoking status, time by MedDiet score, physical activity, energy intake, health status, depression, cognition	**PP:** Usual 20-m walking speed according to the MedDiet score groups, high: referent, low: β-0.014 (SE = 0.02); medium: β-0.008 (SE = 0.002), *p* = 0.049. Rapid 20-m walking speed according to the MedDiet score groups, high: referent, low: β-0.03 (SE = 0.03); medium: β-0.02 (SE = 0.03), *p* < 0.001
S.A. Talegawkar et al. (2012), Italy	InCHIANTI Study Follow-up period: six years (baseline 1998–2000)	Community-living persons (≥65 years) randomly selected from a population registry in Tuscany, Italy Sample size: 690 (333 men, 357 women) Age, Mean (±SD): 73.0 (6.24)	**Tool:** FFQ, validated **DQ:** MDS, ranged from 0 to 9, categorizing into groups: low adherence (MDS ≤ 3); medium adherence (MDS 4–5); high adherence (MDS ≥ 6)	**MS:** Handgrip strength **PP:** Walking speed, 15-feet	Age, sex, energy intake, education, smoking status, BMI, MMSE score, and presence of chronic disease	**MS:** According to the MDS groups, no association **PP:** Low walking speed according to the MDS groups, low: referent, high: OR 0.48 (95% CI = 0.27–0.86)
L.M. León-Muñoz et al. (2014), Spain	Seniors-ENRICA Cohort Follow-up period: 2–4 years (2008–2010 to 2012) Mean follow-up duration: 3.5 years	Community-dwelling individuals aged ≥60 years in Spain Sample size: 1815 Age, Mean (±SE): MEDAS tertile 1 68.5 (0.3); MEDAS tertile 3 68.6 (0.3); MDS tertile 1 68.7 (0.3); MDS tertile 3 68.2 (0.2)	**Tool:** A validated computerized diet history **DQ:** (1) MEDAS, ranged from 0 (lowest) to 14 (highest adherence), cut-off points for tertiles: 6/8 in men, 6/7 in women; (2) MDS, score ranged from 0 (lowest) to 9 (highest adherence), cut-off points for tertiles: 3/5 in men, 3/4 in women	**MS:** Handgrip strength **PP:** Slow walking speed, defined as the lowest quintile in the study sample for the 3-m walking speed test	Sex, age, educational level, tobacco, BMI, energy intake, cardiovascular disease, diabetes mellitus, cancer, asthma or chronic bronchitis, osteo-muscular disease, depression requiring treatment, and number of drug treatments	According to the MEDAS tertiles, **MS:** No association **PP:** Slow walking speed, tertile 1: referent, tertile 3: OR 0.53 (95% CI = 0.35–0.79), *p* for trend: 0.002
						According to the MDS tertiles, **MS:** Weakness as lowest quintile of grip strength, tertile 1: referent, tertile 2: OR 0.69 (95% CI = 0.41–1.16); tertile 3: OR 0.42 (95% CI = 0.24–0.74), *p* for trend: 0.002 **PP:** No association
R. Chan et al. (2016), China	Mr. OS and Ms. OS cohort study Follow-up period: four years (2001–2003 to 2005–2007) Mean follow-up duration: 3.9 years	Chinese men and women aged 65 years or older living in the community Sample size: 2948 for longitudinal analysis (1449 men, 1499 women) Age, Mean (±SD): *	**Tool:** Semi quantitative FFQ, validated **DQ:** MDS, ranges from 0 (minimal) to 9 (maximal adherence)	**Sarcopenia:** AWGS definition	Age, BMI, energy intake, PASE, education level, smoking status, alcohol use, number of chronic diseases, GDS category, CSID category, living alone, and marital status at baseline	**Sarcopenia:** Per ↑ 1point MDS, no association
M. Isanejad et al. (2018), Finland	OSTPRE-FPS Follow-up period: three years	Women aged 65–72 years Sample size: 253 women in the control group only, due to cholecalciferol and calcium supplementation in the intervention group Age, Mean (±SD): by BSD score quartiles, Q1 67.9 (1.8); Q2 67.9 (1.8); Q3 68.0 (1.8); Q4 67.4 (1.8)	**Tool:** A 3-day food record **DQ:** MED score, a score of 8 reflecting maximum adherence, Q1 (≤3); Q2 (4); Q3 (5–6); Q4 (≥7)	**Sarcopenia:** EWGSOP definition **MM:** LM, measured by whole body DXA scans; RSMI, calculated as the sum of the nonfat, nonbone skeletal muscle in arms and legs divided by the square of height **MS:** Handgrip strength **PP:** SPPB; LBMQ, calculated as walking speed 10 m/leg muscle mass	Age, energy intake, smoking, total physical activity, hormone therapy, osteoporosis, rheumatoid arthritis, coronary heart disease, income per month, and fat mass percentage	**MM:** Less lost the total body LM, *p* for trend = 0.001; Less lost the RSMI, *p* for trend = 0.007 based on a linear trend across MED score quartiles MS, PP, **Sarcopenia:** No association
C. H. Huang et al. (2020), Japan	Nagoya Longitudinal Study for Healthy Elderly Follow-up period: three years (2014 to 2017)	Individuals aged over 60 years from a community college in Nagoya, Japan Sample size: 666 (290 men, 376 women) Age, Mean (±SD): 69.4 (4.4)	**Tool:** FFQ, 34 items, adapted from an existing validated FFQ **DQ:** MD, ranged from 0 to 9, with a higher score reflecting greater consistency	**MM:** SMI, obtained by dividing the appendicular muscle mass by squared height, measured by whole-body BIA **MS:** Handgrip strength **PP:** Walking speed, determined according to the participants’ usual gait speed (m/s) over a 5-m course	Age, sex, BMI, educational level, economic status, Charlson Comorbidity Index, Baecke Physical Activity Questionnaire, Mini-Nutritional Assessment, total daily energy, and daily protein intake	**MM, MS, PP:** According to MD tertiles, no association

* Data not provided. InCHIANTI: Invecchiare in Chianti; FFQ: food frequencies questionnaire; DQ: diet quality; MDS: Mediterranean diet score; PP: physical performance; SPPB: short physical performance battery; BMI: body mass index; ADL: activities of daily living; IADL: instrumental activities of daily living; GEE: generalized estimating equations; SE: standard errors; MMSE: mini mental state examination; Health ABC: Health, Aging, and Body Composition; MedDiet: Mediterranean diet; ENRICA: Estudio de Nutrición y Riesgo Cardiovascular; MEDAS: Mediterranean Diet Adherence Screener; MS: muscle strength; OR: odds ratio; CI: confidence interval; AWGS: Asian working group for sarcopenia; PASE: physical activity scale of the elderly; GDS: geriatric depression scale; CSID: cognitive screening instrument for dementia; OSTPRE-FPS: osteoporosis risk factor and prevention-fracture prevention study; BSD: Baltic sea diet; MED: Mediterranean diet; Q: quartile; EWGSOP: European working group on sarcopenia; MM: muscle mass; LM: total body lean mass; DXA: dual-energy X-ray absorptiometry; RSMI: Relative skeletal muscle index; LBMQ: lower body muscle quality; MD: Mediterranean-style diet; BIA: bioelectrical impedance analysis.

**Table 4:** Summary of associations between sarcopenia and diet quality measured by other than the Mediterranean diet.

Author (Year), Country	Cohort, Period	Study Participants	Dietary Assessment	Sarcopenia Assessment	Covariates	Findings
**NDS**						
M.M. Perälä et al. (2016), Finland	Helsinki Birth Cohort Study Follow-up period: 10 years (2001–2004 to 2011–2013)	Participants born as singletons at Helsinki University Central Hospital between 1934–1944 Sample size: 1072 (472 men, 600 women) Age, Mean (±SE): 61.3 (*)	**Tool:** FFQ, validated, 128 items **DQ:** NDS, ranged from 0 to 25 with a higher score indicating greater adherence, based on sex-specific quartiles (NDS <10, 10–12, 13–14, >14)	**PP:** SFT, a modified test battery consists of five measurements: (1) number of chair stands during 30 s; (2) distance walked in 6 min; (3) number of arm curls during 30 s; (4) chair sit and distance between fingers and toe; (5) back scratch (distance between extended middle fingers)	Age, energy intake, BMI, smoking status, educational attainment and physical activity	**PP:** In women, per ↑ 1 point NDS, SFT score: Regression coefficient 0.55 (95% CI = 0.22–0.88); chair stand: Regression coefficient 0.11 (95% CI = 0.03–0.11); 6-min walk: Regression coefficient 0.16 (95% CI = 0.06–0.26); arm curl: Regression coefficient 0.18 (95% CI = 0.09–0.28); chair sit and reach and back scratch: no association. In men, per ↑ 1 point NDS, no association
M. M. Perälä et al. (2017), Finland	Helsinki Birth Cohort Study Follow-up period: 10 years (2001–2004 to 2011–2013)	Participants who were born at Helsinki University Central Hospital between 1934 and 1944 Sample size: 1072 (472 men, 600 women) Age, Mean (±SD): 61.3 (*)	**Tool:** FFQ, validated, 128 items **DQ:** NDS, ranged from 0 to 25 with a higher score indicating greater adherence, based on sex-specific quartiles (NDS <10, 10–12, 13–14, >14)	**MS:** Handgrip strength and leg strength (knee extension) measured in a sitting position **MM:** Muscle mass measured by BIA	Age, energy intake, BMI, smoking status, educational attainment and physical activity	**MS:** In women, per ↑ 1 point NDS, hand grip strength, Regression coefficient 1.44 (95% CI = 0.04–2.84); leg strength, Regression coefficient 1.83 (95% CI = 0.14–3.51). In men, per ↑ 1 point NDS, no association **MM:** In men or women, per ↑ 1 point NDS, no association
M. Isanejad et al. (2018), Finland	OSTPRE-FPS Follow-up period: 3 years	Women aged 65–72 years Sample size: 259 women in the control group only, due to cholecalciferol and calcium supplementation in the intervention group Age, Mean (±SD): by BSD score quartiles, Q1 67.9 (1.8); Q2 67.9 (1.8); Q3 68.0 (1.8); Q4 67.4 (1.8)	**Tool:** A 3-day food record **DQ:** BSD score, ranged from 0 to 25 with a higher score indicating higher adherence, Q1 (≤9); Q2 (10–13); Q3 (14–15); Q4 (≥16)	**Sarcopenia:** EWGSOP definition **MM:** LM, measured by whole body DXA scans; RSMI, calculated as the sum of the nonfat, nonbone skeletal muscle in arms and legs divided by the square of height **MS:** Handgrip strength **PP:** SPPB	Age, energy intake, smoking, total physical activity, hormone therapy, osteoporosis, rheumatoid arthritis, coronary heart disease, income per month and fat mass percentage	**MM:** Less lost the Total body LM, *p* for trend = 0.004; Less lost RSMI, *p* for trend = 0.018 based on a linear trend across BSD score quartiles **PP:** Less decrease of the SPPB, *p* for trend = 0.041 based on a linear trend across BSD score quartiles **MS:** No association
						**Sarcopenia:** The Q4 of BSD score as compared with Q1, OR 0.33 (95% CI: 0.13–0.79), *p* for trend = 0.015 based on a linear trend across BSD score quartiles
**JFG-ST+mJFG-ST+DASH**						
C. H. Huang et al. (2020), Japan	Nagoya Longitudinal Study for Healthy Elderly Follow-up period: three years (2014 to 2017)	Individuals aged over 60 years from a community college in Nagoya, Japan Sample size: 666 (290 men, 376 women) Age, Mean (±SD): 69.4 (4.4)	**Tool:** FFQ, 34 items, adapted from an existing validated FFQ **DQ:** (1) JFG-ST, ranged from 0 to 60; (2) mJFG-ST, ranged from 0 to 70; (3) DASH, ranged from 6 (minimal adherence) to 30 (maximal adherence); was determined by tertiles	**MM:** SMI, obtained by dividing the appendicular muscle mass by squared height, measured by whole-body BIA **MS:** Handgrip strength **PP:** Walking speed, determined according to the participants’ usual gait speed (m/s) over a 5-m course	Age, sex, BMI, educational level, economic status, Charlson Comorbidity Index, Baecke Physical Activity Questionnaire, Mini-Nutritional Assessment, total daily energy, and daily protein intake	According to JFG-ST tertiles, **MM:** SMI, Q1: referent, Q3: mean difference 0.048 (0.002–0.095), *p* = 0.040 In men, Q2: mean difference 0.098, *p* = 0.047; Q3: mean difference 0.091, *p* = 0.017. In women, no association MS, **PP:** No association
						According to mJFG-ST and DASH tertiles, MM, MS, **PP:** No association
**DVS**						
N. Kojima et al. (2015), Japan	A comprehensive health checkup conducted by the Tokyo Metropolitan Institute of Gerontology Follow-up period: four years (baseline 2008)	Community-dwelling women aged 75–85 years from the Itabashi Ward of Tokyo Sample size: 575 women Age, Mean (±SD): 78.07 (2.56)	**Tool:** Interview by closed-ended questions **DQ:** DVS, which is an index of dietary variety of the 10 food groups, ranges from 0 (consuming once/two days or less for all of the food groups) to 10 (consuming almost every day for all of the food groups)	**MS:** Knee extension strength	Baseline age, baseline knee extensor strength, and baseline status of all the diseases (hypertension, stroke, heart disease, diabetes mellitus, hyperlipidemia, osteoporosis, anemia, asthma, COPD, hip osteoarthritis, gonarthrosis)	**MS:** According to DVS ≤ 5 and DVS ≥ 6 groups, no association
Y. Yokoyama et al. (2017), Japan	Kusatsu Longitudinal Study, Hatoyama Cohort Study Follow-up period: 4 years (2010 to 2014)	(1) The Kusatsu Longitudinal Study: 65 years or older living in the town of Kusatsu; (2) The Hatoyama Cohort Study: Community-dwelling adults aged 65 years or older living in the town of Hatoyama Sample size: 779 (416 men, 363 women) Age, Mean (±SD): by DVS group, Lowest 71.1 (4.9); Middle 71.8 (5.1); Highest 72.8 (5.6)	**Tool:** Interview on consumption frequencies for each of 10 food items of DVS **DQ:** DVS, assessing dietary quality based on a count of the number of 10 food items consumed, categorized into three groups: Lowest (DVS = 0–3); Middle (DVS = 4–6); Highest (DVS ≥ 7)	**MM:** Muscle mass measured by Multifrequency BIA **MS:** Handgrip strength **PP:** Gait speed, measured over a straight 11-m walkway	Age, sex, education (years), living status, self-perceived chewing ability, smoking habit, drinking habit, exercise habit, BMI, depressive symptoms, cognitive status, and physician-diagnosed illnesses	**MS:** Decline in handgrip strength, lowest: referent, highest: OR 0.43 (95% CI = 0.19–0.99), *p* for trend = 0.049 based on a linear trend across DVS tertiles **PP:** Decline in usual gait speed, lowest: referent, highest: OR 0.43 (95% CI = 0.19–0.99), *p* for trend = 0.039 based on a linear trend across DVS tertiles **MM:** No association
**DQI-I**						
R. Chan et al. (2016), China	Mr. OS and Ms. OS cohort study Follow-up period: four years (2001–2003 to 2005–2007) Mean follow-up duration: 3.9 years	Chinese men and women aged 65 years or older living in the community Sample size: 2948 for longitudinal analysis (1449 men, 1499 women) Age, Mean (±SD): *	**Tool:** Semi quantitative FFQ, validated **DQ:** DQI-I, ranges from 0 to 94 and higher score indicates better diet quality	**Sarcopenia:** AWGS definition	Age, BMI, energy intake, PASE, education level, smoking status, alcohol use, number of chronic diseases, GDS category, CSID category, living alone, and marital status at baseline	**Sarcopenia:** Per ↑ 1point DQI-I score, no association
**Factor analysis**						
L.M. León-Muñoz et al. (2015). Spain	Seniors-ENRICA cohort Follow-up period: 2–4 years (2008–2010 to 2012) Mean follow-up duration: 3.5 years	Non-institutionalized individuals aged ≥60 years Sample size: 1872 (906 men, 966 women) Age, Mean (±SE): 68.7 (*)	**Tool:** A validated computerized diet history **DQ:** Factor analysis, (1) prudent pattern; (2) Westernized pattern, a higher score indicated a higher adherence	**MS:** Handgrip strength **PP:** Slow walking speed, the lowest quintile in the study sample for the 3-m walking speed test	Number of frailty components at baseline, sex, age, educational level, occupation, tobacco, BMI, energy intake, cardiovascular disease, diabetes mellitus, cancer, asthma or chronic bronchitis, osteomuscular disease, depression requiring treatment, number of drug treatments, and score on the MMSE	According to the prudent pattern tertiles, **MS, PP:** No association
						According to the Westernized pattern tertiles, **PP:** Slow walking speed, tertile 1: referent, tertile 3: OR 1.85 (95% CI = 1.19–2.87), *p* for trend: 0.007 **MS:** No association
R. Chan et al. (2016), China	Mr. OS and Ms. OS cohort study Follow-up period: four years (2001–2003 to 2005–2007) Mean follow-up duration: 3.9 years	Chinese men and women aged 65 years or older living in the community Sample size: 2948 for longitudinal analysis (1449 men, 1499 women) Age, Mean (±SD): *	**Tool:** Semi quantitative FFQ, validated **DQ:** Factor analysis, (1) ‘vegetables-fruits’; (2) ‘snacks-drinks-milk products’; (3) ‘meat-fish’; a higher score represented greater conformity	**Sarcopenia:** AWGS definition	Age, BMI, energy intake, PASE, education level, smoking status, alcohol use, number of chronic diseases, GDS category, CSID category, living alone, and marital status at baseline	**Sarcopenia:** Per ↑ 1 point dietary pattern scores, no association
A. Granic et al. (2016), UK	The Newcastle 85+ Study Follow-up period: five years (baseline 2006)	Very old adults (aged 85+) born in 1921, who lived in Newcastle and North Tyneside, UK Sample size: 791 (302 men, 489 women) Age: 85	**Tool:** 24-hr multiple pass dietary recall, validated **DQ:** Cluster analysis, (1) DP1 ‘High Red Meat’; (2) DP2 ‘Low Meat’; (3) DP3 ‘High Butter’	**MS:** Handgrip strength **PP:** TUG test	Sex, education, dominant hand, diet change in the past year and health-related variables (season-specific serum vitamin D quartiles, total energy, number of chronic diseases, BMI), lifestyle variables (physical activity, smoking)	**MS:** Handgrip strength, DP2: referent, DP1: β-0.92 (SE = 0.46), *p* = 0.05. In men, DP1: β-1.70 (SE = 0.86), *p* = 0.05. In women, no association **PP:** TUG test, DP2: referent, DP3: β 0.05 (SE = 0.02), *p* = 0.002. In men, DP1: β 0.08 (SE = 0.02), *p* = 0.001. In women, DP3: β 0.06 (SE = 0.02), *p* = 0.01
A. Granic et al. (2020), UK	The Newcastle 85+ Study Follow-up period: three years (baseline 2006)	Community-dwelling older adults aged ≥85 years of 1921 birth cohort in Newcastle and North Tyneside, UK Sample size: 757 (294 men, 463 women) Age: 85	**Tool:** 24 h multiple pass dietary recall, validated **DQ:** Cluster analysis, (1) DP1 ‘Low Red Meat’; (2) DP2 ‘Traditional British’; (3) DP3 ‘Low Butter’	**Sarcopenia:** EWGSOP definition	BMI, socio-demographic factors (sex, social class, education), health-related factors (cognitive status, depressive symptoms, total number of diseases, total number of medications), lifestyle factors (physical activity, smoking, food energy)	**Sarcopenia:** Prevalent sarcopenia, DP3: referent, DP2: OR 2.42 (95% CI=1.15–5.09), *p* = 0.02. In the good protein intake group ( ≥ 1 g/kg aBW/day), DP3: referent, DP2: OR 5.45 (95% CI = 1.8–16.36), *p* = 0.003

* Data not provided. FFQ: food frequencies questionnaire; DQ: diet quality; NDS: Nordic diet score; PP: physical performance; MS: muscle strength; MM: muscle mass; BIA: bioelectrical impedance analysis; BMI: body mass index; SFT: the senior fitness test; OSTPRE-FPS: osteoporosis risk factor and prevention-fracture prevention study; BSD: Baltic sea diet; Q: quartile; EWGSOP: European working group on sarcopenia; LM: total body lean mass; DXA: dual-energy X-ray absorptiometry; RSMI: relative skeletal muscle index; SPPB: short physical performance battery; OR: odds ratio; CI: confidence interval; JFG-ST: Japanese food guide spinning top; mJFG-ST: modified JFG-ST; DASH: Dietary Approaches to Stop Hypertension; DVS: dietary variety score; COPD: chronic obstructive pulmonary disease; DQI-I: Diet quality index-international; AWGS: Asian working group for sarcopenia; PASE: physical activity scale of the elderly; GDS: geriatric depression scale; CSID: cognitive screening instrument for dementia; ENRICA: Estudio de Nutrición y Riesgo Cardiovascular; UK: United Kingdom; DP: dietary pattern; TUG: timed up-and-go; SE: standard errors.

## Data Availability

No new data were created or analyzed in this study. Data sharing is not applicable to this article.

## Supplementary Materials

The following are available online at https://www.mdpi.com/article/10.3390/life11080811/s1, Table S1: Search terms.
