# Supplementary material for: Association between Diet Quality and Sarcopenia in Older Adults: Systematic Review of Prospective Cohort Studies
Source: Life (Basel). 2021 Aug 10;11(8):811. doi: 10.3390/life11080811 (PMC8399213; doi:10.3390/life11080811)
Supplement: Supplementary file 1 [file life-11-00811-s001.zip › life-1310318-supplementary.pdf]

Table S1. Search terms.

**Pubmed**	
1	"sarcopenia"[all fields]
2	"sarcopeni*"[title/abstract]
3	"frail*"[title/abstract]
4	"muscle*"[title/abstract]
5	"muscle atrophy"[all fields]
6	"muscular atrophy"[all fields]
7	"muscle mass"[all fields]
8	"muscle weak*"[all fields]
9	"Lean body mass"[all fields]
10	"fat free mass"[all fields]
11	"Body composition"[all fields]
12	"muscle strength"[all fields]
13	"grip strength"[all fields]
14	"hand strength"[all fields]
15	"physical performance"[all fields]
16	"physical function*"[all fields]
17	"aging phenotype*"[all fields]
18	"mobility "[all fields]
19	"disability"[all fields]
20	"gait"[all fields]
21	"walking"[all fields]
22	"timed get up and go"[all fields]
23	1 OR 2 OR 3 OR 4 OR 5 OR 6 OR 7 OR 8 OR 9 OR 10 OR 11 OR 12 OR 13 OR 14 OR 15 OR 16 OR 17 OR 18 OR 19 OR 20 OR 21 OR 22
24	"diet"[all fields]
25	"diet*"[title/abstract]
26	"diets"[all fields]
27	"food habit*"[all fields]
28	"healthy eating"[title/abstract]
29	"healthful eating"[title/abstract]
30	"eating habit*"[title/abstract]
31	"eating pattern*"[title/abstract]
32	"food habit*"[title/abstract]
33	"dietary pattern*"[title/abstract]
34	"dietary quality"[title/abstract]
35	"dietary adherence"[title/abstract]
36	"food pattern*"[title/abstract]
37	"Mediterranean"[title/abstract]
38	"dietary score"[title/abstract]
39	"meal"[title/abstract]
40	24 OR 25 OR 26 OR 27 OR 28 OR 29 OR 30 OR 31 OR 32 OR 33 OR 34 OR 35 OR 36 OR 37 OR 38 OR 39
41	"old"[all fields]
42	"older*"[all fields]
43	"elder*"[all fields]
44	"senior*"[all fields]
45	"aging"[all fields]
46	41 OR 42 OR 43 OR 44 OR 45
47	23 AND 40 AND 46, Filter: MEDLINE, human, and English
**EMBASE**	

1	sarcopenia OR sarcopeni*:ab,ti OR frail*:ab,ti OR muscle*:ab,ti OR 'muscle atrophy' OR 'muscular atrophy' OR 'muscle mass' OR 'muscle weak*' OR 'lean body mass' OR 'fat free mass' OR 'body composition' OR 'muscle strength' OR 'grip strength' OR 'hand strength' OR 'physical performance' OR 'physical function*' OR 'aging phenotype*' OR mobility OR disability OR gait OR walking OR 'timed get up and go'
2	diet OR diet*:ab,ti OR diets OR 'food habit*' OR 'healthy eating':ab,ti OR 'healthful eating':ab,ti OR 'eating habit*':ab,ti OR 'eating pattern*':ab,ti OR 'food habit*':ab,ti OR 'dietary pattern*':ab,ti OR 'dietary quality':ab,ti OR 'dietary adherence':ab,ti OR 'food pattern*':ab,ti OR mediterranean:ab,ti OR 'dietary score':ab,ti OR meal:ab,ti
3	old OR older* OR elder* OR senior* OR aging
4	1 AND 2 AND 3 AND [1-1-1947]/sd NOT [1-12-2020]/sd
